# Aberrant Intraregional Brain Activity and Functional Connectivity in Patients With Diarrhea-Predominant Irritable Bowel Syndrome

**DOI:** 10.3389/fnins.2021.721822

**Published:** 2021-09-03

**Authors:** Xiao-Fei Chen, Yun Guo, Xing-Qi Lu, Le Qi, Kuang-Hui Xu, Yong Chen, Guo-Xiong Li, Jian-Ping Ding, Jie Li

**Affiliations:** ^1^Department of Radiology, The Affiliated Hospital of Hangzhou Normal University, Hangzhou, China; ^2^Department of Gastroenterology, The Affiliated Hospital of Hangzhou Normal University, Hangzhou, China; ^3^Medical College, Hangzhou Normal University, Hangzhou, China

**Keywords:** irritable bowel syndrome, amplitude of low-frequency fluctuation, regional homogeneity, functional connectivity, resting-state fMRI

## Abstract

**Background and Purpose:**

The appearance and aggravation of diarrhea-predominant irritable bowel syndrome (IBS-D) have proven to be closely related to psychosocial factors. We aimed to measure altered spontaneous brain activity and functional connectivity (FC) in patients with IBS-D using resting-state functional magnetic resonance imaging (RS-fMRI) and to analyze the relationship between these parameters and emotional symptoms.

**Methods:**

Thirty-six adult IBS-D patients and thirty-six demographic-matched healthy controls (HCs) underwent RS-fMRI scans. After processing RS-fMRI data, the values of the amplitude of low-frequency fluctuation (ALFF) and regional homogeneity (ReHo) of the two groups were compared. The abnormal regions were selected as the regions of interest to compare whole-brain seed-based FC between the groups. The relationships between RS-fMRI data and mood and gastrointestinal symptoms were analyzed using correlation and mediation analyses.

**Results:**

Compared with HCs, IBS-D patients showed increased ALFF in the right cerebellum posterior lobe, the right lingual gyrus/calcarine, the right postcentral gyrus, the right superior frontal gyrus (SFG), and middle frontal gyrus (MFG), with decreased ALFF in the right inferior parietal lobule, the right striatum, the right anterior cingulated cortex, the right insula, the right hippocampus, the right thalamus, the right midbrain, and the left precuneus. IBS-D patients showed increased ReHo in the bilateral lingual gyrus/calcarine, the bilateral SFG, the right MFG, and the right postcentral gyrus, with decreased ReHo in the orbital part of the left inferior frontal gyrus and the right supplementary motor area. Patients showed enhanced FC between the left precuneus and the bilateral orbitofrontal cortex (OFC). There was a positive correlation between increased ALFF values in the right midbrain and anxiety-depression symptoms in IBS-D patients, and the mediating effect of gastrointestinal symptoms indirectly caused this correlation.

**Conclusion:**

IBS-D patients had dysregulated spontaneous activity and FC in regions related to pain regulation and emotional arousal involved in prefrontal–limbic–midbrain circuit and somatosensory processing. The development of mood disorders in IBS-D patients may be partly related to the dysfunction of components in the dopamine pathway (especially the midbrain, OFC) due to visceral pain.

## Introduction

Irritable bowel syndrome (IBS) is one of the most common functional gastrointestinal disorders (FGID), characterized by recurrent abdominal pain or discomfort and changes in bowel habits (Mearin et al., 2016). IBS is classified into diarrhea type (IBS-D), constipation type (IBS-C), mixed type, and undefined type according to the predominant stool pattern. Patients with IBS-D account for about one-third of all patients, and their quality of life is lower than that of other subtypes; their daily activities are more disturbed, and they commonly avoid eating (Rome Foundation, 2006; Singh et al., 2015). IBS is often accompanied by psychological disorders, among which anxiety and depression are the most common, with a prevalence of up to 50% (Ford et al., 2020). Patients with IBS-D have loose stools and frequent bowel movements, especially accompanied by urgency and fear of urinary incontinence, making them more prone to panic and anxiety (Singh et al., 2015). Treatment options for IBS-D are limited (Lucak et al., 2017; Dothel et al., 2018).

The pathogenesis of IBS is not entirely understood; however, it is thought to be related to visceral hypersensitivity, psychosocial factors, intestinal infections, and intestinal flora disturbance (Ford et al., 2020). Psychosocial factors have recently attracted particular attention because of their essential role in the brain-gut axis. Specifically, psychosocial factors affect the central processing of visceral stimulation through the emotional circuit of the central nervous system, leading to further amplification of central pain in a top-down approach (Mudyanadzo et al., 2018). Patients with IBS have insufficient secretion of neurotransmitters related to gastrointestinal function [e.g., 5-hydroxytryptamine (5-HT) and dopamine], which affects the operation of neurotransmitter-related circuits in the brain from bottom to top, further aggravating emotional disorders (Gershon and Tack, 2007; Stasi et al., 2012). Nevertheless, the exact neuropathological mechanisms of IBS-induced emotional disturbance remain unclear. Psychosocial factors might impact pain thresholds differently according to the subtype of IBS. Affective and emotional symptoms should be considered specific and parts of the syndrome. Recognizing their impact on different IBS subtypes may have implications for treatment options and clinical outcomes (de Medeiros et al., 2008; Muscatello et al., 2010).

Functional magnetic resonance imaging (fMRI) is a powerful tool for evaluating brain function by detecting relative changes in the content of local deoxyhemoglobin when measuring neuronal activity, including in task-fMRI and resting-state fMRI (RS-fMRI) experimental modes. During rectal dilation, the brain regions associated with the fronto–limbic circuit for regulating emotion, particularly the insula, the amygdala, and the prefrontal cortex (PFC), were abnormally activated in patients with IBS (Elsenbruch et al., 2010; Labus et al., 2013; Hong et al., 2016). RS-fMRI can detect spontaneous neural activity without specific tasks with a high spatial resolution (Fox and Raichle, 2007). RS-fMRI studies showed that IBS patients have abnormal spontaneous activities in brain regions related to pain processing [i.e., the amygdala, anterior cingulated cortex (ACC), thalamus, and insula] and emotion regulation (i.e., the amygdala, cingulate cortex, hippocampus, and hypothalamus), and partly attributable to anxiety and depression (Ma et al., 2015; Qi et al., 2016). This association between abnormalities in brain activity and psychological symptoms was analyzed by comparing outcomes with and without the inclusion of anxiety and depression symptoms as covariates. Based on independent component analysis or graph theory analysis, IBS was found to be associated with dysfunction of functional connectivity (FC) in several networks, including the salience/executive control network, the attention network, the emotion regulation network, and the default mode network (DMN) (Mayer et al., 2019). Few fMRI studies on IBS-specific subtypes and only two task-fMRI studies observed differences in brain activation patterns between IBS-C and IBS-D when receiving rectal dilation stimulation. The activation of the pain matrix related brain regions in IBS-D patients was higher than that in IBS-C patients and was related to the higher sensitivity of IBS-D patients to rectal dilation (Wilder-Smith et al., 2004; Guleria et al., 2017). Therefore, it is necessary to observe the patterns of brain function changes during resting-state and their correlation with emotional symptoms in patients with specific subtypes of IBS.

The amplitude of low-frequency fluctuations (ALFF) (Zang et al., 2007; Zuo et al., 2010) and regional homogeneity (ReHo) (Zang et al., 2004) are commonly used RS-fMRI methods for local neural activity. The ALFF measures the regional intensity of a voxel in a low-frequency range (Zang et al., 2007; Zuo et al., 2010). The ReHo calculated the synchronization of low-frequency fluctuations between a given voxel with neighboring voxels (Zang et al., 2004). Therefore, ALFF and ReHo complement each other and provide a more comprehensive characterization of local brain abnormalities (An et al., 2013; Lv et al., 2019; Li et al., 2021).

The functional brain patterns of IBS-D patients during resting-state and their relationship to emotional symptoms remain unclear. Based on previous research and theoretical results, we selected IBS-D patients as the research subjects, utilized ALFF and ReHo methods for data-driven analysis, and combined with FC analysis to explore changes in whole-brain spontaneous neural activity and FC in patients with IBS-D. We hypothesized that there would be aberrant brain function in emotion or pain-related processing regions in IBS-D patients, and the partly neuroimaging changes would be associated with emotional symptoms. In particular, gastrointestinal manifestations with pain as the core symptom may mediate this correlation.

## Materials and Methods

Thirty-eight patients with IBS-D and 36 age-, sex- and education level- matched healthy controls (HCs) were recruited from the Gastroenterology Clinic and Physical Examination Center of the Affiliated Hospital of Hangzhou Normal University from June 2016 to July 2018. All patients were diagnosed simultaneously by two senior gastroenterologists. All subjects provided written informed consent before participating. The Research Ethics Committee of the Affiliated Hospital of Hangzhou Normal University approved the study that we performed according to the Declaration of Helsinki of the World Medical Association.

All patients with IBS-D underwent a complete medical history, physical examination, blood biochemistry, and colonoscopy. The inclusion criteria included age 18–55 years and meeting the Rome III diagnostic criteria. The exclusion criteria included taking antidepressants and gastrointestinal motility drugs in the previous 2 weeks, a history or current diagnosis of psychosocial disorders (e.g., major depression, psychosis, or drug and alcohol abuse as defined by the DSM-IV-TR criteria), a history of brain trauma or craniocerebral surgery, and pregnancy or lactation. The age range of HCs was 18–55 years, and the exclusion criteria were the same as those of the IBS-D patients. All research subjects were right-handed according to the Edinburgh Hand-Handling Scale.

### Questionnaire Scale Assessment

Before the MRI, the clinical data of all IBS patients were collected, including the course of the disease and the gastrointestinal symptom rating scale (GSRS) (Svedlund et al., 1988). The GSRS scale includes 15 common gastrointestinal symptoms, and each item is divided into seven levels (range 1–7 points), and the total score range of the scale is 15–105 points. All subjects completed the 17-item Hamilton Depression Rating Scale (HAMD-17) (Hamilton, 1960) and the 14-item Hamilton Anxiety Rating Scale (HAMA-14) (Thompson, 2015) questionnaires under the guidance of two psychiatrists. These two questionnaires are the most used scales for clinical evaluation of depression and anxiety. Scores greater than seven suggest depression or anxiety. Before and after the scan, the visual analog scale (VAS) (Huskisson, 1974) was used to evaluate the patient’s pain intensity at baseline and during the scan.

### MRI Data Acquisition

GE Discovery-750 3.0T MRI (GE Discovery MR-750, Milwaukee, WI) and 8-channel head coil were used for data acquisition. Subjects are required to lie on their backs on the MRI scanning bed, wear special non-magnetic headphones, and use sponge cushions to fix both sides of the head to reduce head movement. During the image acquisition process, the participants need to remain awake, relax with their eyes closed, breathe smoothly, and keep their heads motionless as much as possible. The scanning sequences included the whole-brain three-dimensional high-resolution T1 weighted image (3D T1WI) obtained through the three-dimensional spoiled gradient recalled-echo sequence and the RS-fMRI image obtained by the blood oxygenation level dependent-gradient echo-echo planar imaging sequence. The parameters of 3D T1WI are repetition time (TR) = 8.16 ms, echo time (TE) = 3.18 ms, flip angle = 8°, the field of view (FOV) = 256 mm × 256 mm, Acquisition matrix (matrix) = 256 × 256, layer thickness = l mm, 176 layers. The scan parameters of RS-fMRI are TR = 2,000 ms, TE = 30 ms, flip angle = 90°, FOV = 192 × 192 mm, Matrix = 64 × 64, layer thickness = 4 mm, interval = 0 mm. The scan range includes the whole brain, and the scan time is 8 min in total. T1WI and T2 fluid-attenuated inversion recovery were collected simultaneously to rule out abnormal anatomical structures and organic brain diseases by visual inspection; subjects with abnormal signs such as intracranial space-occupying lesions and cerebral infarction were excluded.

### MRI Data Preprocessing

Data preprocessing was implemented using the Data Processing and Analysis for (Resting-State) Brain Imaging (DPABI) software package,^1^ which is based on Statistical Parametric Mapping version 8 (SPM 8).^2^ Data were converted from the DICOM format to the NIFTI format using the MRI CONVERT software.^3^ The first ten volumes were deleted to achieve signal balance and participant adaptation to the scan. Then slice-timing and head motion correction were performed. Subjects with head motion exceeding 3.0 mm translation or 3.0 rotation in any direction were excluded. Each T1WI was co-registered to the mean motion-corrected function images. We used DARTEL (Diffeomorphic Anatomical Registration using Exponentiated Lie algebra) for registration, normalization, and modulation (Ashburner, 2007). A customized template was generated using the average tissue probability maps across all participants, and the segmented map from each participant was warped into the template. This procedure was repeated until the best study-specific template was generated. The images were then modulated based on Jacobian determinants. Thereafter, the functional images were registered to the Montreal Neurological Institute (MNI) space using the registration information of the structural image (resampling voxel size = 3 mm × 3 mm × 3 mm). Finally, spatial smoothing with a Gaussian kernel with 6-mm full-width at half-maximum (FWHM) (smoothing was applied after ReHo calculation). Nuisance covariates were regressed out, including the head movement parameters, cerebrospinal fluid signals, and white matter using the Friston 24-parameter model. Temporal band-pass filtering (0.01–0.08 Hz) was performed for ReHo and FC analyses.

### ALFF, ReHo, and FC Calculation

The time series of a given voxel was converted into a frequency domain using the fast Fourier transform. The ALFF value was calculated by obtaining the square root of the power spectrum and then averaging it in the range of 0.01–0.08 Hz. The ALFF of each voxel was divided by the global mean ALFF value to standardize data across subjects.

Kendall’s coefficient of concordance was used to calculate the local synchronization between a voxel and the nearest 26 voxels. The standardized ReHo map was then obtained by dividing the ReHo value of each voxel by the global mean ReHo value (Zang et al., 2004). Spatial smoothing (FWHM = 6 mm) was then performed.

FC analysis was performed to investigate the connectivity patterns between the regions of interest (ROI) and the voxels of the rest brain. Based on the results of ALFF and ReHo analysis, the changed brain regions [FWE-corrected with threshold-free cluster enhancement (TFCE), *P* < 0.05] in IBS-D were chosen as the ROIs (spherical regions with a radius of 5 mm centered on the peak coordinates), including the right lingual gyrus, the right superior frontal gyrus (SFG), the right postcentral gyrus, the right inferior parietal lobule (IPL), the right supplementary motor area (SMA), the right cerebellar posterior lobe, the right supramarginal gyrus, the right midbrain, the orbital part of the left inferior frontal gyrus (orb_IFG), and the left precuneus. The mean time series of each ROI was extracted, Pearson’s correlation coefficients were calculated between the mean time series of each ROI and each voxel’s time series of the whole brain to generate a correlation map. Finally, Fisher’s *r*-to-*z* transform was performed to improve the normality of the correlation coefficient.

### Statistical Analysis

SPSS software version 18 (SPSS, Chicago, Illinois, United States) was adopted to analyzed demographic and clinical data. Two-sample *t*-tests were used to explore the ALFF, ReHo, and FC differences between the two groups. Age, sex, education level, and mean head movement parameters were used as covariates in these comparisons. Multiple comparison correction with TFCE was performed with 5,000 permutation tests (*P* < 0.05 after correction) (Smith and Nichols, 2009). Partial correlation analyses were then conducted between altered ALFF values, ReHo values, FC *z*-values and mood/gastrointestinal symptoms in the IBS-D group. Gender, age, and education level were included as covariates, and *P* < 0.05 was considered statistically significant. If the correlations were significant, a tentative bootstrapped mediation analysis was performed using the PROCESS macro in SPSS (Hayes, 2013) to explore whether the abnormal ALFF/ReHo in a specific brain region had a direct or indirect effect on the emotional symptoms (or gastrointestinal symptoms) through the gastrointestinal symptoms (or emotional symptoms). Age, gender, and years of education were taken as covariables. The significance was estimated using a bias-corrected bootstrapping method with 5,000 iterations, and *P* < 0.05 was considered statistically significant.

## Results

### Demographic Characteristics and Clinical Results

Two patients were excluded due to excessive head movement, leaving 36 IBS-D patients and 36 HCs for further analysis. Demographic details of all participants and their clinical characteristics are summarized in Table 1.

**TABLE 1:** Demographic and clinical characteristics of participants.

Variables	IBS-D (*n* = 36)	HCs (*n* = 36)	*t*-values	*P*-values
Sex(male/female)	16/20	10/26	2.17	0.15^a^
Age (years)	34.36 ± 9.53	31.67 ± 8.85	1.24	0.22^b^
Education (years)	12.08 ± 3.12	13.28 ± 3.32	–1.57	0.12^b^
HAMD-17	7.33 ± 4.60	0.42 ± 0.77	8.90	<0.001^b^
HAMA-14	8.92 ± 5.80	1.19 ± 1.70	7.67	<0.001^b^
Duration of IBS (months)	19.11 ± 6.98	–	–	–
GSRS	30.50 ± 12.78	–	–	–
Before scan -VAS	3.69 ± 1.95	–	–	–
During scan -VAS	4.72 ± 2.19	–	–	–

*Data are shown as mean ± standard deviation.^a^ χ^2^; ^b^ independent-sample t-test, two-tailed. IBS-D, diarrhea-predominant irritable bowel syndrome; HCs, healthy controls; HAMD, Hamilton depressive scale; HAMA, Hamilton anxiety scale; GSRS, gastrointestinal symptoms rating scale; VAS, visual analog scale.*

There were no significant differences between the groups concerning sex (*t* = 2.17, *P* = 0.15), age (*t* = 1.24, *P* = 0.22), or education level (*t* = –1.57, *P* = 0.12). Mean disease duration of IBS-D patients was 19.11 months. Mean GSRS degree was 30.5. The depression and anxiety scores of patients were higher than those of HCs (*t* = 8.09, *P* < 0.001; *t* = 6.96, *P* < 0.001). The VAS score of patients during the MRI scan was greater than the score before the scan (*t* = –2.10, *P* = 0.04).

### fMRI Data Analyses

**FIGURE 1:**
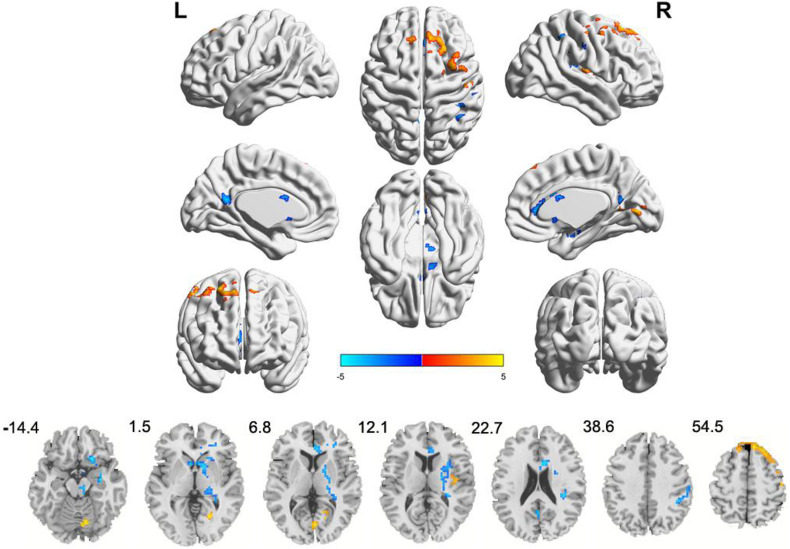
Clusters showing ALFF changes in IBS-D patients as compared to that in healthy controls (TFCE, FWE-corrected *P* < 0.05). Blue color denotes relatively lower ALFF values in IBS-D group, red color denotes relatively higher ALFF values in IBS-D group, and the color bar in dicates the *t*-value from two-sample *t*-test between IBS-D group and HCs. L, left; R, right.

**FIGURE 2:**
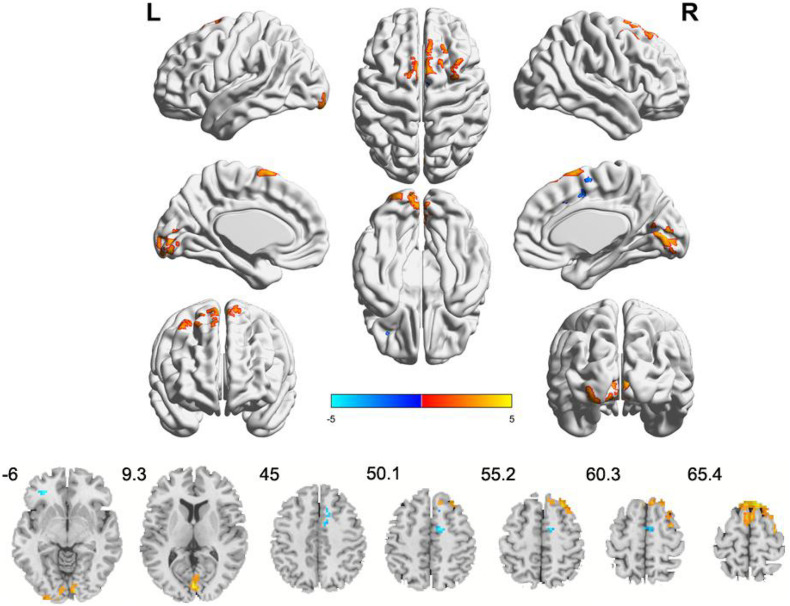
Clusters showing ReHo changes in IBS-D patients as compared to that in healthy controls (TFCE, FWE-corrected *P* < 0.05). Blue color denotes relatively lower ReHo values in IBS-D group, red color denotes relatively higher ReHo values in IBS-D group, and the color bar in dicates the *t*-value from two-sample *t*-test between IBS-D group and HCs. L, left; R, right.

**FIGURE 3:**
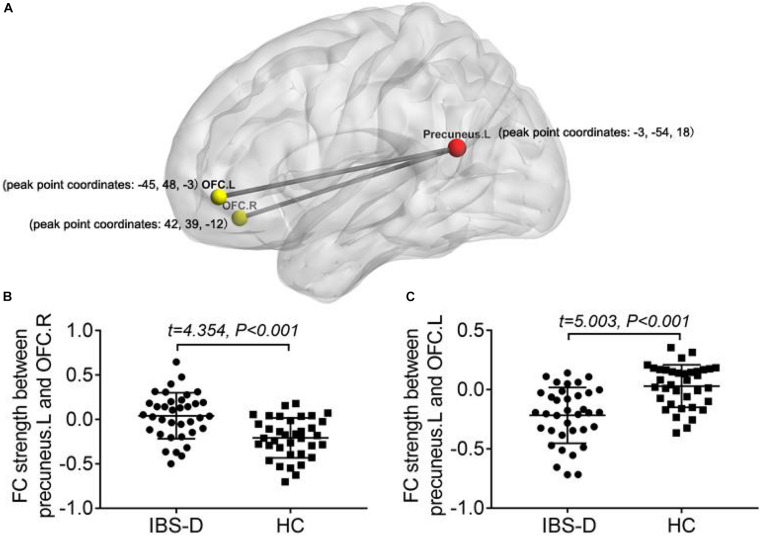
Comparing to HCs, IBS-D patients showed enhanced connectivity between the seed region in the left precuneus and the bilateral orbital frontal cortex (TFCE, FWE-corrected *P* < 0.05) **(A)**. Distribution of FC values between left precuneus and right OFC **(B)**/left OFC **(C)** in the two groups and differences between groups. L, left; R, right; FC, functional connectivity; OFC, orbital frontal cortex; IBS-D, diarrhea-predominant irritable bowel syndrome; HC, healthy controls.

Compared to the HCs, IBS-D patients had significantly higher ALFF values in the right cerebellum posterior lobe, the right lingual gyrus/calcarine, the right postcentral gyrus, the right SFG, and the right MFG, with lower ALFF values in the right IPL, the right striatum, the right ACC, the right insula, the right hippocampus, the right thalamus, the right midbrain, and the left precuneus (Figure 1 and Table 2). IBS-D patients had higher ReHo values in the bilateral lingual gyrus/calcarine, the bilateral SFG, the right MFG, and the right postcentral gyrus, with lower ReHo values in the left orb_IFG and the right SMA (Figure 2 and Table 3). Compared to the HCs, IBS-D patients showed enhanced connectivity between the left precuneus [the peak point coordinates was (–3, –54, 18)] and the bilateral orbitofrontal cortex (OFC) (right OFC: the peak point coordinates was [42, 39, –12], the peak *t*-value was 4.262, and the number of voxels was 31; left OFC: The peak point coordinates was [–45, 48, –3], the peak *t*-value was 4.809, and the number of voxels was 86) (Figure 3). These brain regions were described according to the Anatomical Automatic Labeling (AAL) templates. We also conducted ROI-based whole-brain FC analysis with other different brain regions as ROI, and no significant difference was found after multiple comparison corrections between the groups.

**TABLE 2:** Brain regions with abnormal ALFF in IBS-D patients compared to HCs.

MNI coordinates of maximum voxel			Cluster size (voxels)	BA	Peak voxel *t*-value	Anatomical region
*x*	*y*	*z*				
**IBS-D > HC**						
12	–72	–15	17		4.325	Right cerebellum posterior lobe
21	–63	0	18	30	4.725	Right lingual gyrus
3	–75	6	22	18/30	4.384	Right calcarine
51	–15	54	16	3	4.673	Right postcentral gyrus
9	33	63	304	6/8	4.974	Right superior and middle frontal gyrus (peak region: right medial superior frontal gyrus)
**IBS-D < HC**						
42	–39	36	730	13/24/40	–4.939	Right putamen, right supramarginal gyrus, right caudate, right anterior cingulated gyrus, right insula, right hippocampus, right thalamus, right pallidum, left caudate (peak region: right supramarginal gyrus)
6	–21	–12	22		–3.400	Right midbrain
–3	–54	18	40	23/31	–4.026	Left precuneus
42	–42	48	23	40	–3.899	Right inferior parietal lobule

*IBS-D, diarrhea-predominant irritable bowel syndrome; HCs, healthy controls; MNI, Montreal Neurological Institute; BA, Brodmann area.*

**TABLE 3:** Brain regions with abnormal ReHo in IBS-D patients compared to HCs.

MNI coordinates of maximum voxel			Cluster size (voxels)	BA	Peak voxel *t*-value	Anatomical region
*x*	*y*	*z*				
**IBS-D > HC**						
9	–84	–3	236	18/17	4.555	Bilateral calcarine, bilateral lingual gyrus (peak region: right lingual gyrus)
9	30	63	276	6/8	5.073	Bilateral superior frontal gyrus, right middle frontal gyrus(peak region: right medial superior frontal gyrus)
33	0	63	19	6	4.058	Right superior frontal gyrus
30	–30	66	15	3	4.417	Right postcentral gyrus
**IBS-D < HC**						
–30	39	–6	16	47	–4.489	Left inferior frontal gyrus (orbital part)
15	–3	51	50	6	–4.651	Right supplementary motor area

*IBS-D, diarrhea-predominant irritable bowel syndrome; HCs, healthy controls; MNI, Montreal Neurological Institute; BA, Brodmann area.*

### Correlation and Mediation Analyses

**FIGURE 4:**
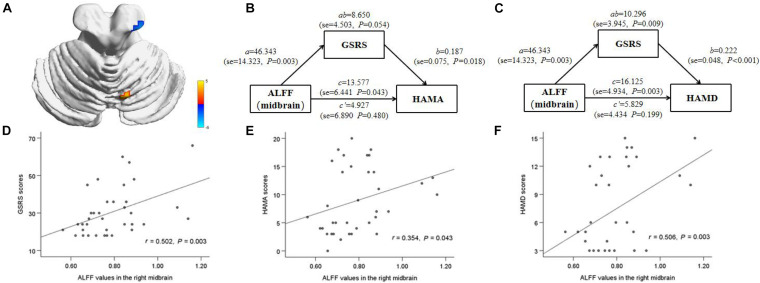
Correlation and mediation analyses between ALFF value in the right midbrain and emotional symptoms. The area in blue color shows a reduction in ALFF (midbrain) in IBS-D patients compared to the healthy controls **(A)**. The gastrointestinal symptoms completely mediated the relationship between ALFF in the right midbrain and HAMA (critical statistical significance), HAMD scale **(B,C)**. The positive correlations were observed between ALFF value in right midbrain and gastrointestinal symptoms and HAMA, HAMD scale in IBS-D patients **(D–F)**. HAMD, Hamilton depressive scale; HAMA, Hamilton anxiety scale; GSRS, gastrointestinal symptoms rating scale.

In IBS-D patients, ALFF values in the right midbrain positively correlated with HAMD, HAMA, and GSRS score (*r* = 0.506, *P* = 0.003; *r* = 0.349, *P* = 0.047; *r* = 0.501, *P* = 0.003). Mediation analysis revealed that the ALFF value of the right midbrain had a significant overall effect on anxiety (critical statistical significance, *P* = 0.054) and depression (*P* = 0.009) (Models 1 and 2) and was completely mediated by gastrointestinal symptoms. The statistical values of the specific mediation model were as follows: Model 1 Path c, *P* = 0.028; Path a, *P* = 0.002; Path b, *P* < 0.001; Path c, not significant; Standardization indirect effect = 22.932, 95% confidence interval: 8.238, 43.578. Model 2 Path c, *P* = 0.006; Path a, *P* = 0.002; Path b, *P* < 0.001; Path c’, not significant; Standardization indirect effect = 15.118, 95% confidence interval: 5.086, 24.785 (Figure 4). These results suggest that ALFF in the right midbrain tends to have an indirect effect on psychological symptoms predominantly through visceral pain.

## Discussion

To the best of our knowledge, this is the first study applying ALFF, ReHo, and FC simultaneously to detect abnormalities of spontaneous brain activity in IBS-D patients, and explored the correlations between these abnormalities and clinical variables. We identified significant regional brain functional alterations in the PFC (mainly the bilateral dorsolateral prefrontal cortex [DLPFC]), the limbic system (including the right hippocampus, the right striatum, the right thalamus, the right insula, and the right ACC), the midbrain, the sensorimotor cortex, and the visual cortex. Based on the abnormal brain regions in ALFF and ReHo, FC analysis showed enhanced connectivity between the left precuneus (seed region) and the bilateral OFC. These findings have implications for understanding the functional mechanisms of IBS-D and its related mood disorders.

### Altered Brain Activity Implicated in Regions of the Pain Regulation and Emotional Arousal Within the Prefrontal–Limbic–Midbrain Circuit in IBS-D Patients

### Prefrontal Cortex

The PFC is essential for higher-order cognitive, language, and emotional processing (Wiech et al., 2008). IBS symptoms characterized by chronic visceral pain can be manifested at several levels, including visceral hyperalgesia and lowered pain threshold in the sensory dimension, emotional symptoms such as anxiety and depression induced by chronic pain, and cognitive dysfunction such as decreased learning ability and memory loss. The sensory and emotional abnormalities caused by pain are regulated by cognitive function. Cognitive regulation of pain can be categorized as attention, expectation, and reevaluation (Wiech et al., 2008), all of which involve different regions of the PFC, especially the DLPFC and OFC (Jensen et al., 2012).

As the core component of the central control network, the DLPFC plays a regulatory role in pain perception through the descending inhibitory pathway (Lorenz et al., 2003). Our previous and other brain morphometric studies reported atrophied cortical thickness or gray matter volume of DLPFC in patients with IBS and other chronic pain syndromes (Schmidt-Wilcke et al., 2006; Hubbard et al., 2016; Li et al., 2020). The DLPFC participates in “keeping pain out of mind,” especially in chronic pain states (Lorenz et al., 2003; Wiech et al., 2008). The higher pain scores of patients undergoing MRI in this study may be related to the amplification of pain perception during the scan. In addition, the ALFF and ReHo values of DLPFC in IBS-D patients showed a consistent increase, further suggesting that DLPFC plays a vital role in visceral hypersensitivity and the reduction of pain threshold.

OFC is thought to play a vital role in adjusting emotions, decision-making, pain adjustment, and the integration of visceral sensorimotor information (Kringelbach and Rolls, 2004; Price, 2007; Moont et al., 2011). Jarcho et al. (2008) found that activity in the bilateral OFC could be used to predict IBS symptom improvement by 5-HT subtype 3 general receptor antagonists. A study combining positron emission tomography (PET) and RS-fMRI found that dopamine receptor availability was higher in individuals with more robust connectivity between OFC and DMN (Cole et al., 2012). The left precuneus is the core node of the DMN, which maintains self-awareness and episode-memory retrieval during the resting state (Cavanna and Trimble, 2006; Zhang and Li, 2012). IBS patients showed activation of the precuneus and adjacent brain regions under painful rectal dilation stimulation (Kano et al., 2017). The present study showed that IBS-D patients had the abnormal local neural activity of OFC and the precuneus, as well as enhanced FC between the bilateral OFC and the precuneus, which we speculated might be related to the dysfunction of the dopamine pathway (such as changes in dopamine receptor availability), further leading to the corresponding mood and pain symptoms.

### Limbic System

The insula and ACC belong to the medial system of the pain matrix and are associated with the affective-motivational component, which is essential for visceral sensation, autonomous visceral movement, pain and emotional processing (Nakata et al., 2014). These two regions have been the most consistently abnormal functional and structural regions in IBS patients and were significantly associated with negative emotions (Blankstein et al., 2010; Jiang et al., 2013; Piché et al., 2013; Qi et al., 2016; Kano et al., 2020). The hippocampus is the region involved in memory formation, memory organization, and memory storing, inhibiting feedback on the hypothalamic-pituitary-adrenal axis (Tasker and Herman, 2011). IBS patients have abnormal hippocampus activation and dysregulation of the hippocampal-amygdala circuit when performing cognitive tasks (Aizawa et al., 2012). Niddam et al. (2011) found a decreased concentration of glutamate complex of the left hippocampus in IBS patients and negatively correlated with anxiety. The striatum is a vital nucleus of the basal ganglia and a core element of the pain matrix, which is involved in the integration of information in the cortex, thalamus and three specific pain processing regions (sensory, emotional/cognitive, and endogenous/regulatory) (Legrain et al., 2011). Our previous study showed that the reduction of gray matter volume in the ACC, insula, and basal ganglia was significantly associated with visceral hypersensitivity in patients with IBS, which was highly consistent with other studies (Bhatt et al., 2019; Li et al., 2020). This study found that patients with IBS-D have decreased ALFF values in regions of the limbic system involved in pain regulation and pain-related emotional processing, including ACC, insula, hippocampus, basal ganglia, and thalamus, which may be related to the long-term visceral hypersensitivity, visceral pain, emotional and cognitive impairment.

### Midbrain

The periaqueductal gray matter (PAG) and dorsal raphe nucleus play essential roles in the descending pain inhibition pathway at the midbrain level, which may be related to the activation of dopamine neurons and the high expression of opioid receptors (Takada et al., 2013; Li et al., 2016). Tominaga et al. (2015) found that upregulation of serotonin transporter levels in the midbrain and thalamus may underlie the pathogenesis of FGID such as abdominal and psychological symptoms through brain-gut interactions. In a quantitative meta-analysis of task-fMRI studies related to rectal stimulation, IBS subjects showed consistent activation of a large midbrain region (Tillisch et al., 2011). Consistent with this, our study showed that decreased ALFF values in the midbrain during the resting state and was positively correlated with abdominal and psychological symptoms in IBS-D patients. By applying mediation analysis, we found that the relationship between ALFF in the right midbrain and psychological symptoms was entirely mediated by abdominal symptoms, although the analysis of anxiety symptoms was only statistically critical significant. These results are consistent with previous studies on pathogenesis biology, suggesting that primary intestinal disorders may be the underlying driving factors of mood disorders (Jones et al., 2012; Koloski et al., 2012). The decrease of ALFF in the right midbrain might indicate the dysfunction of the descending pain inhibitory pathway in IBS-D patients, leading to visceral hypersensitivity and abdominal pain, and further aggravating emotional symptoms.

### Altered Brain Activity Implicated in Somatic and Sensory Processing in Patients With IBS-D

The postcentral gyrus belongs to the parietal lobe, also known as the somatosensory center, part of the pain matrix. It may play a crucial role in adjusting pain perception, including the positioning and recognition of pain intensity. Our finding of increased ALFF and ReHo in the postcentral gyrus paralleled that reported by Ke et al. (2015), resulting from adaptive neuronal changes caused by long-term pain stimuli and biological responses. The SMA is part of the sensorimotor region that receives information from the basal ganglia. It is also thought to be part of the ventral prefrontal parietal attention network, which is involved in detecting and directing attention to behavior-related sensory stimuli while ignoring irrelevant, competitive stimuli (Vossel et al., 2014). An RS-fMRI study suggested that SMA may play a role in suppressing the abnormal emotional effects caused by physical symptoms such as abdominal distension (Zhu et al., 2016). The abnormal local neural activity in the brain areas related to somatic and sensory processing (e.g., posterior central gyrus and SMA) may be closely related to the degree of pain and visceral hypersensitivity in patients with chronic visceral pain, and affect emotional and cognitive functions to a certain extent.

Our study also found increased local activity in the cerebellum posterior lobe and the visual cortex. The cerebellum is one of the elements of the pain matrix, which can transmit pain information upward through the fiber connection with the cerebral cortex and participate in pain regulation by affecting the downward inhibitory pathway of the brainstem nociceptive (Naegel et al., 2014). Transcranial cerebellar direct current stimulation can effectively regulate the perception and reception of pain (Wiech et al., 2010; Bocci et al., 2019). The occipital cortex is traditionally regarded as a region related to visual information processing. However, neuroimaging studies reported changes in the signal intensity of the occipital lobe in patients with chronic pain (Klug et al., 2011; Li et al., 2018). A task-fMRI study found that IBS patients showed increased activity in the visual cortex during the expectation of rectal pain, suggesting that it is easier to promote the attention process under uncertain conditions (Lee et al., 2012). Increased local neural activity in these two posterior brain regions in IBS-D patients may be associated with hypervigilance for pain perception and regulation.

Although both ReHo and ALFF analysis methods are reliable means to assess the functional activity of the brain, most of the brain regions obtained by these two methods in this study did not overlap, except for the right DLPFC, the right postcentral gyrus, and the right lingual gyrus. These results suggest that, in addition to the abnormal activity of local neurons in these regions during the resting state of IBS-D patients, more of these region-related neurons are involved. The difference in results between the two methods may indicate different forms of functional dysfunction in neuronal activity in multiple brain regions in IBS-D, which may also contribute to the complexity of clinical manifestations of the disease.

A few limitations should be noted in this study. First, the sample size was small. Second, a longitudinal investigation is needed to verify the indirect effect of functional activity in brain regions such as the midbrain as a possible cause of IBS-D on emotional scores. Future studies will explore the reliability and reproducibility of these results across multiple cohorts for test-retest.

## Conclusion

By combining ReHo, ALFF, and FC analysis, we found abnormal spontaneous activity and FC dysregulation in areas related to pain regulation and emotional arousal in the prefrontal–limbic–midbrain circuit and areas related to somatosensory processing. The development of mood disorders in IBS-D patients may be related to the dysfunction of dopamine pathway components (especially midbrain and OFC) caused by visceral pain.

## Data Availability Statement

The raw data supporting the conclusions of this article will be made available by the authors, without undue reservation.

## Ethics Statement

The studies involving human participants were reviewed and approved by the Research Ethics Committee of the Affiliated Hospital of Hangzhou Normal University. The patients/participants provided their written informed consent to participate in this study.

## Author Contributions

X-FC and JL designed and supervised the project. YG, X-QL, K-HX, YC, G-XL, and LQ collected the data. X-FC, J-PD, and JL processed and analyzed the data. All authors revised the manuscript and approved the final version.

## Conflict of Interest

The authors declare that the research was conducted in the absence of any commercial or financial relationships that could be construed as a potential conflict of interest.

## Publisher’s Note

All claims expressed in this article are solely those of the authors and do not necessarily represent those of their affiliated organizations, or those of the publisher, the editors and the reviewers. Any product that may be evaluated in this article, or claim that may be made by its manufacturer, is not guaranteed or endorsed by the publisher.

## Footnotes

1
http://rfmri.org/DPABI

2
https://www.fil.ion.ucl.ac.uk/spm/software/spm8/

3
http://lcni.uoregon.edu/~jolinda/MRIConvert/
